# Polyphenols of *Frangula alnus* and *Peganum harmala* Leaves and Associated Biological Activities

**DOI:** 10.3390/plants9091086

**Published:** 2020-08-24

**Authors:** Hosam O. Elansary, Agnieszka Szopa, Paweł Kubica, Halina Ekiert, Fahed A. Al-Mana, Ahmed A. El-Shafei

**Affiliations:** 1 Plant Production Department, College of Food and Agricultural Sciences, King Saud University, Riyadh 11451, Saudi Arabia; falmana@ksu.edu.sa; 2 Floriculture, Ornamental Horticulture, and Garden Design Department, Faculty of Agriculture (El-Shatby), Alexandria University, Alexandria 21545, Egypt; 3 Department of Geography, Environmental Management, and Energy Studies, University of Johannesburg, APK campus, Johannesburg 2006, South Africa; 4 Department of Pharmaceutical Botany, Medical College, Jagiellonian University, 30-688 Medyczna, Poland; p.kubica@uj.edu.pl (P.K.); halina.ekiert@uj.edu.pl (H.E.); 5 Department of Agricultural Engineering, College of Food and Agriculture Sciences, King Saud University, Riyadh 11451, Saudi Arabia; aelshafei1bn.c@ksu.edu.sa; 6 Agricultural and Biosystems Engineering Department, Faculty of Agriculture, Alexandria University, Alexandria 21545, Egypt

**Keywords:** *Frangula alnus*, *Peganum harmala*, antioxidant, antiproliferative, cytotoxicity, caspase, H_2_O_2_, polyphenols

## Abstract

*Frangula alnus* and *Peganum harmala* populations growing in Saudi Arabia might be rich sources of natural compounds with important biological activities. A high performance liquid chromatography diode array revealed several polyphenols in the leaf extracts for the first time, including *p*-coumaric acid, rosmarinic acid, chlorogenic acid, ferulic acid, quercitrin, rutoside, quercetin and trifolin in *F. alnus;* and hydrocaffeic acid, protocatechuic acid, rosmarinic acid, caffeic acid and cynaroside in *P. harmala. F. alnus* and *P. harmala* showed strong antioxidant effects attributed to the polyphenolic composition of leaves and reduction of reactive oxygen species (ROS) accumulation. *F. alnus* and *P. harmala* leaf extracts showed cytotoxic effects against Jurkat, MCF-7, HeLa, and HT-29 cancer cells using MTT and flow cytometry assays. These activities were attributed to the polyphenolic composition of leaves including quercitrin, trifolin and cymaroside, as well as the activation of caspase family enzymes 2, 6, 8 and 9 in treated cancer cells compared to control. The current findings of this study include a novel comprehensive investigation on the polyphenol composition and anticancer effects of leaf extracts of *F. alnus* and *P. harmala* from natural populations in Saudi Arabia.

## 1. Introduction

Natural populations of medicinal plants have been a valuable source of therapeutic compounds and drug discovery [1]. These compounds include polyphenols which may reduce certain age-associated illnesses including cancer and Alzheimer’s disease by confronting escalated cellular damage through significant reduction of reactive oxygen species (ROS) and inflammatory status [2,3]. The antiproliferative and cytotoxic activities of polyphenols against human cancer cells are also attributed to cell cycle arrest and intermolecular regulation of known genes [4,5,6,7,8,9]. 

*Frangula alnus* (Rhamnaceae) is a tall shrub growing in western Asia, Europe and northernmost Africa [10]. The shrub bark is dark, with a bright lemon-yellow inner bark, and is the main economic part of the plant. The bark is widely used in traditional as well as modern medicine as laxative (Council of Europe, 2008). These laxative properties of the bark have been attributed to frangulins, anthraquinone glycoside derivatives, anthraquinone monoglycosides (emodin) and glucofrangulins components [11,12]. For example, the bark extract of *F. alnus* (Croatian origin) showed moderate antioxidant effects attributed to emodin [12]. Another study revealed that the bark extracts had strong antibacterial activities against *Staphyllococcus aureus* [13]. From our knowledge, no studies have been conducted on the biological activity and chemical components of the leaves of *F. alnus* natural populations. Previous investigation showed that this species is invasive and can survive and adapt in different environmental conditions including wetlands [14]. Indeed, these environmental conditions (e.g., temperature and humidity) affect the plant’s physiology and secondary metabolite production [15].

*Peganum harmala* (Zygophyllaceae) is a medicinal and ornamental plant naturally growing in the southern eastern parts of Asia and Northern Africa [16]. In traditional medicine, *P. harmala* seeds are used as decoctions, powder and infusion for diarrhea, abortion, asthma, lumbago and other ailments [16]. Most previous investigations on *P. harmala* studied seed composition and reported high antioxidant activities [17]. Little attention was given to the chemical composition of the leaves, which showed antioxidant and antibacterial activities [18,19]. However, no specific polyphenols were identified. Other compounds were identified in the seeds such as vasicinone, harmine, peganine and harmalacidine, which have antiproliferative and cytotoxic effects against Med-mek carcinoma, UCP-med sarcoma and Jurkat [20,21]. The Chinese origin *P. harmala* showed high amounts of 4-hydroxyisoleucine, asparagine, proline, lysine, vasicine and sucrose in methanolic extracts of leaves [22]. *P. harmala* grows in Saudi Arabia as natural populations and no antioxidant, cytotoxic or antimicrobial studies were conducted on these populations. Previous investigation on Algerian *P. harmala* showed that environmental conditions may influence the distribution of this species in salted areas [23]. Indeed, these conditions may affect the chemical composition of this species.

*F. alnus* and *P. harmala* are natural medicinal plants growing in many parts of Saudi Arabia and collected for medicinal uses by local healers. Both species are drought tolerant and grow naturally in similar environmental conditions. The current study is a novel work on the polyphenol content and biological effects of the leaf extracts of *F. alnus* and *P. harmala* from populations in Saudi Arabia. 

## 2. Materials and Methods 

### 2.1. Sampling of Plant Material 

Leaves of *Frangula alnus* Mill. and *Peganum harmala* L. were obtained from populations growing naturally in the Riyadh region of Saudi Arabia. Identification and vouchering were conducted at King Saud University (College of Food and Agricultural Sciences, Hosam0002215-103) by Hosam Elansary. The samples were collected in May 2019 at a temperature ranging from 33 to 23 °C and humidity ranging from 17 to 20%. Sandy soils are common in the collection site, which is close to Al-Thumamah National Park (25°07′ 15″ N 46° 40′ 13″ E, altitude is 632 m above sea level). Methanolic leaf extracts were prepared following [24].

### 2.2. Phenolic Compounds

A liquid chromatograph (Merck-Hitachi, LaChrom Elite) containing a DAD detector (L-2455) was used for the chromatographic analyses. A Purospher RP-18e column (250 × 4 mm; 5 μm, Merck) was used with a mobile phase: A—methanol, and B—0.5% acetic acid and methanol 1:4 (v/v), in a gradient program: 0–20′ 100% B; 20–35′ 100–80% B; 35–55′ 80–60% B; 55–70′ 60–0% B; 70–75′ 0%; 0–100% B for 75–80′; 80–90′ 100% B (1 ml/min). The injection volume of the sample was 20 µL, temperature was set at 25 °C and wavelengths were from 210 to 400 nm. Qualitative and quantitative analyses were read at 254 nm. The method was validated former by our group [25,26]. Retention times and UV spectra were compared to standards to identify the compounds investigated in the methanolic extracts. Calibration curves were used to detect the chemical compounds. Calibration equations of detected compounds were as follows: chlorogenic acid (y = 44790418.3x − 45222.5), caffeic acid (y = 59811859.9x − 145632.3), *p*-coumaric acid (y = 31143463.3x + 37623.6), ferulic acid (y = 60949159.6x + 44967.3), rutoside (y = 59420774.2x + 66560.0), quercetin (y = 67269930.4x − 813339.4), rosmarinic acid (y = 36295528.6x − 232028.2), quercitrin (y = 57911264.5x − 1446794.6), trifolin (y = 80650805.9x − 583711.0), protocatechuic acid (y = 135776068.2x − 259871.0), hydrocaffeic acid (y = 5883705.0x + 30869.7), cynaroside (y = 100536033.3x +- 3483461.8). All 38 standards used for analyses were obtained from Sigma-Aldrich (Berin, Germany). We screened for 22 phenolic acids including: benzoic acid and some derivatives (ellagic, 3,4-dihydroxyphenylacetic, protocatechuic, *p*-hydroxybenzoic, gallic, vanillic, gentisic, syringic and salicylic acids); cinnamic acid and some derivatives (sinapic, *o*-, *m*-, *p*-coumaric acid, isoferulic, ferulic, hydrocaffeic and caffeic acids); and depsides (neochlorogenic, rosmarinic and chlorogenic acids); seven flavonoid aglycones (naringin, quercetin, cynaroside, rhamnetin, luteolin, myricetin and kaempferol); and nine flavonoid glycosides (trifolin, vitexin, apigetrin, cynaroside, robinin, rutoside, hyperoside, quercitrin and isoquercetin). 

### 2.3. Antiproliferative Effects

Antiproliferative effects of *F. alnus* and *P. harmala* extracts were tested against an array of cancer cells including Jurkat, HeLa, MCF-7, and HT-29. [6,27,28]. HEK-293 normal human cells were also used. To measure the changes in cell viability (antiproliferative effect), we used MTT. The leaf extracts obtained before were solubilized in DMSO (1%). Leaf extract solutions (serial concentrations) were added to prepared MEM medium containing 0.1 mM nonessential amino acids, 10% FBS, 1 mM sodium pyruvate, and 17.8 mM NaHCO_3_ in 75 cm^2^ flasks. The medium contained cancer/normal cells 4 × 10^−4^ cells µL^-1^. A washing step was performed using PBS. The MTT solution (12 mM) was mixed the medium. Isopropanol (0.04 N HCl) was mixed as well and left for 40 min. A positive control (vinblastine sulfate and taxol) and negative control (untreated) were used. Absorbance was measured at 570 nm wavelength using the following equation:

$$The\;inhibition\;activity\;percentage\;=\;\frac{{\left(AB_{570\;\mathrm{nm}}\right)}_{C}-{\left(AB_{570\;\mathrm{nm}}\right)}_{s}}{{\left(AB_{570\;\mathrm{nm}}\right)}_{C}}\times 100$$

where:

AB is absorbance
${\left(AB_{570nm}\right)}_{C}$ and ${\left(AB_{570nm}\right)}_{s}$ are the absorbances of the control and sample, respectively.

### 2.4. Cytotoxic Effects 

The IC_50_ values of each prepared extract were calculated by plotting percentage of viable cells against the concentration of the extract in µg mL^−1^. These IC_50_ values were employed in the flow cytometry experiment. The apoptotic cell populations were determined for selected cancer cells using an FAC Scan, USA [6,27,29].

### 2.5. Antioxidant Activity 

The antioxidant effects of *F. alnus* and *P. harmala* extracts were explored in three different experiments: ferric reducing antioxidant power (FRAP), β-carotene bleaching and 2,2-diphenyl-1-picrylhydrazyl (DPPH) [28,30,31,32,33,34]. The IC_50_ (µg/mL) values were defined as the amount of extract scavenging 50% of β-carotene bleaching/DPPH solution/FRAP reagent. These values were calculated by plotting the inhibition percent against extract concentration. In the DPPH experiment, serial concentrations of the extracts were incubated in methanolic DPPH solution previously prepared (5 mL of 0.004%) for 30 min in the dark at room temperature. The absorbance was measured at 517 nm. A positive control was used in the experiment (butylated hydroxytoluene, BHT). In the β-carotene-bleaching experiment, the absorbance was measured at 470 nm. In the FRAP experiment, Trolox (positive control) was used and the absorbance was measured at 593 nm. All experiments were repeated thrice.

### 2.6. ROS Intercellular Accumulation

This assay determined the ability of the obtained leaf extracts to reduce the intracellular levels of ROS in HeLa, Jurkat, T24, and MCF-7 cancer cells. The assay employs the fluorogenic dye (H_2_DCF-DA) [35]. The cancer cells were exposed to leaf extracts or polyphenols IC_50_ values (determined by DPPH). DCF fluorescence was determined after 90 min of treatment at 485 nm. Hydrogen peroxide (H_2_O_2_) was used as a positive control.

### 2.7. Caspase Activity by Colorimetric Assay

The effect of *F. alnus* and *P. harmala* extracts on caspase activity in cancer cells was determined using the Protease Sampler Kit (Invitrogen, Carlsbad, CA). The cells were cultured for 1 d in RPMI growth medium containing the IC_50_ of the extracts/polyphenols, then harvested and tested for caspase activity according to the protocol of the manufacturer. Briefly, control and treated cells were resuspended in chilled cell lysis buffer (50 mL) then incubated on ice for 10 min. The lysates were centrifuged for 1 min (10,000 g). The concentration of the protein was calculated using Bradford’s method. The reaction buffer was added to the protein and incubated 2 h at 37°C. The reaction buffer contained 200 mM substrate VEID (caspase-6), IETD-pNA (caspase-8), LEHD-pNA (caspase-9), and VDVAD-pNA (caspase-2). Absorbance was measured at 405 nm. The relative caspase activity (expressed as % of untreated control) was determined by the comparison of the absorbance of pNA from an apoptotic sample with the control.

### 2.8. Statistical Analyses

Least significant difference (LSD) was calculated by using SAS software. The values are means ± SDs of three series of experiments.

## 3. Results

### 3.1. F. alnus and P. harmala Polyphenol Profiling of Leaf Extracts

**Figure 1:**
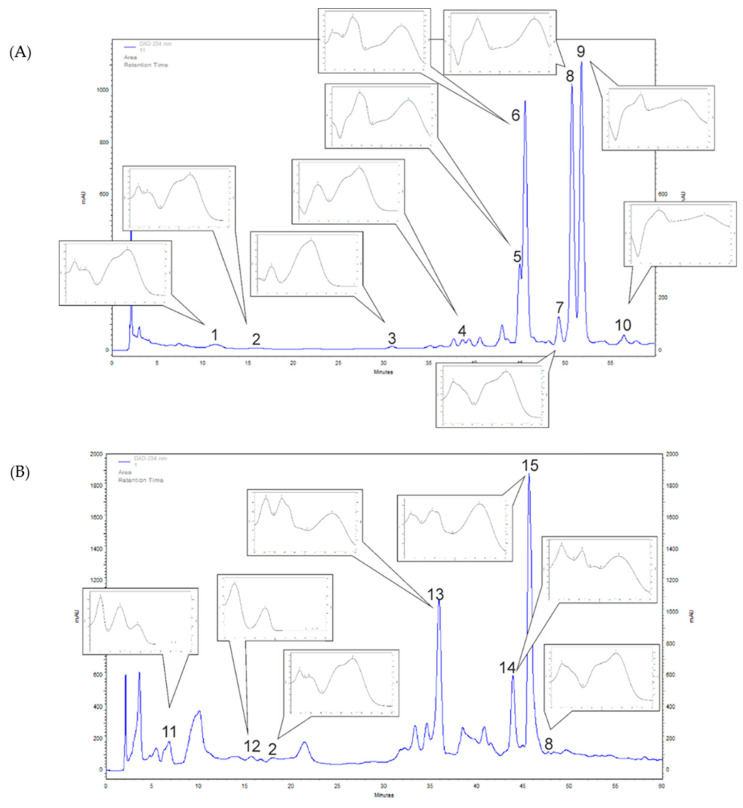
Samples of high performance liquid chromatography diode array (HPLC-DAD) separation (λ = 254 nm) of the extracts of: (**A**) *F. alnus* and (**B**) *P. harmala* (1—chlorogenic acid, 2—caffeic acid, 3—p-coumaric acid, 4—ferulic acid, 5—rutoside, 6—unknown compound, 7—quercetin, 8—rosmarinic acid, 9—quercitrin, 10—trifolin, 11—protocatehuic acid, 12—hydrocaffeic acid, 13—unknown compound, 14—unknown compound, 15—cynaroside).

**Figure 2:**
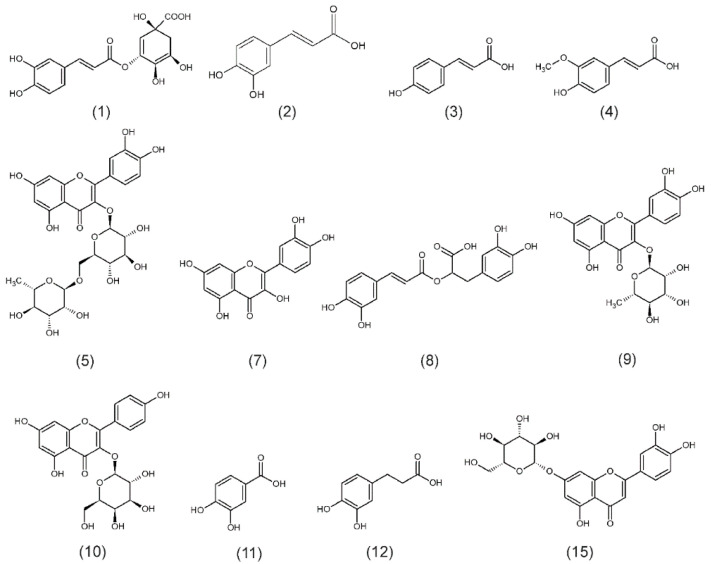
The chemical structures of the identified compounds: 1—chlorogenic acid, 2—caffeic acid, 3—*p*-coumaric acid, 4—ferulic acid, 5—rutoside, 7—quercetin, 8—rosmarinic acid, 9—quercitrin, 10—trifolin, 11—protocatehuic acid, 12—hydrocaffeic acid, 15—cynaroside.

The HPLC-DAD analyses of *F. alnus* extracts revealed nine phenolic compounds from phenolic acids (chlorogenic (1), caffeic (2), p-coumaric (3), ferulic (4) and rosmarinic (8) acids), and flavonoids (quercitrin (9), rutoside (5), quercetin (7) and trifolin (10)) (Figure 1A and Figure 2). From phenolic acids, the highest amounts were confirmed for rosmarinic acid (269.5 mg/100 g DW (dry weight)) and chlorogenic acid (126.8 mg/100 g DW) (Table 1). From flavonoids, the amounts of compounds were: quercitrin (1132.3 mg/100 g DW), trifolin (914.3 mg/100 g DW), rutoside (283.4 mg/100 g DW) and quercetin (115.1 mg/100 g DW) (Table 1). In the *F. alnus* extract, an abundant unknown compound (6) was recognized, based on UV spectra, as a flavonoid derivative (Figure 1A). Its amount was calculated as quercitrin equivalent (the dominant flavonoid in *F. alnus* extract), and was 1072.4 mg/100 g DW (Table 1).

In *P. harmala* methanolic leaf extracts, four phenolic acids (protocatehuic (11), hydrocaffeic (12), caffeic (2), rosmarinic (8) acids) and one flavonoid, cynaroside (15) were identifed (Figure 1B and Figure 2). A significant amount of hydrocaffeic acid (199.4 mg/100 g DW) and protocatechiuc acid (51.6 mg/100 g DW) were detected (Table 1). The amounts of caffeic and rosmarinic acids were much lower (20.6 and 18.9 mg/100 g DW, respectively) (Table 1). The determined cynaroside content was high and amounted to 713.5 mg/100 g DW (Table 1). In the *P. harmala* extract, UV spectra indicated that two more abundant flavonoid derivatives were found (compounds 13 and 14) as shown in Figure 1B. Their amounts were 503.8 and 254.0 mg/100 g DW, respectively, as cynaroside equivalent (Table 1).

### 3.2. Antioxidant Effects

*F. alnus* and *P harmala* extracts showed strong antioxidant effects comparable to selected polyphenols as shown in Table 2. *F. alnus* showed significantly higher antioxidant effects than *P. harmala* as detected by different assays including *β*-carotene bleaching DPPH and FRAP. *F. alnus* polyphenols such as trifolin, *p*-coumaric acid, and rosmarinic acid showed strong antioxidant effects (low IC_50_). *P. harmala* polyphenols including hydrocaffeic acid, cynaroside and protocatechuic acid showed strong antioxidant effects. Rosmarinic acid antioxidant activities were comparable to antioxidant standards.

### 3.3. MTT Assay 

The antiproliferative effects of *F. alnus* and *P. harmala* extracts against selected cancer cells were evaluated using the MTT test (Table 3). There were antiproliferative effects of *F. alnus and P. harmala* extracts, as well as selected polyphenols, against all cancer cells. The normal cells of HEK-293 were not affected by the extracts. *F. alnus* showed higher antiproliferative activities than *P. harmala.* Noticeable antiproliferative effects were found when applying polyphenols such as quercitrin, cynaroside, trifolin, *p*-coumaric acid and rutoside against cancer cells. 

### 3.4. Flow Cytometry

**Figure 3:**
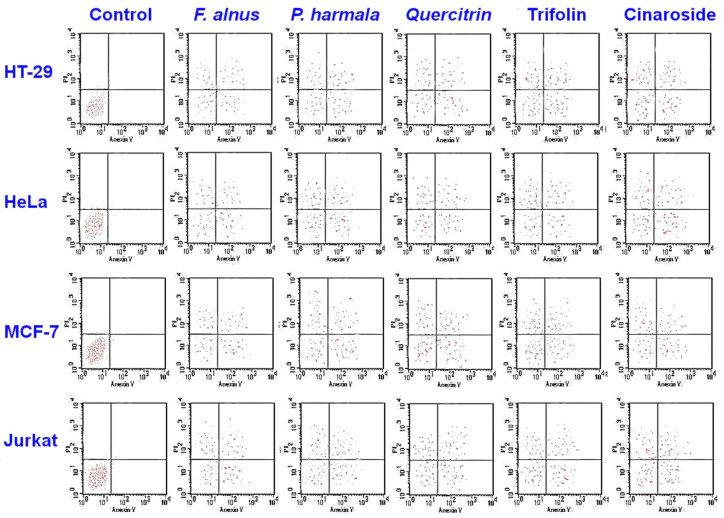
Cytotoxicity of *F. alnus* and *P. harmala* methanolic extracts, quercitrin, trifolin, and cynaroside, as estimated by flow cytometry. Lower left, viable cells; upper left, necrotic cells; lower right, early apoptotic cells and upper right, late apoptotic cells. Flow cytometry showed obvious apoptotic cell accumulation following 48 h of exposure in the upper and lower right quadrant.

The cytotoxic activities of *F alnus* and *P. harmala* extracts, as well as quercitrin, trifolin, and cymaroside, were studied using the flow cytometry technique (Figure 3). The experiments showed obvious apoptotic cell accumulation following 2 d of exposure in the upper and lower right quadrant. 

### 3.5. ROS Accumulation Assay 

**Figure 4:**
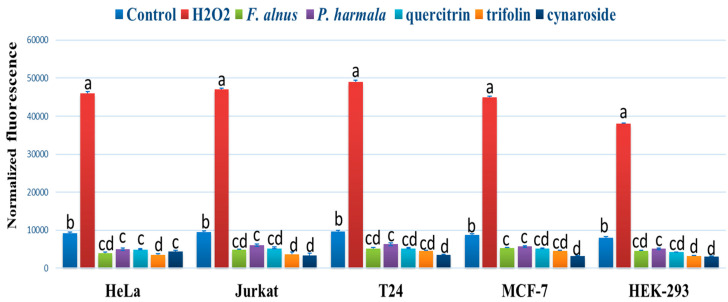
The effect of *F. alnus* and *P. harmala* methanolic extracts, quercitrin, trifolin, and cynaroside DPPH IC_50_ on the DCF fluorescence in HeLa, Jurkat, T24, MCF-7, and HEK-293 cells. Medium with 1 mM hydrogen peroxide (H_2_O_2_) was used as a positive control. The highest reduction of ROS was achieved using trifolin and cynaroside in all cancer cells following 90 min of incubation. The data are expressed as the mean ± SD of three experiments (four replicates per treatment). Different letters among columns in specific cells indicate significant differences at *p ≤ 0.05*.

*F. alnus* and *P. harmala* extracts, as well as quercitrin, trifolin, and cynaroside, reduced the accumulation of ROS in treated cells compared to control using H_2_DCFDA fluorescence (Figure 4). The highest reduction of ROS was found when using trifolin, and cynaroside in all cells after 90 min of incubation. H_2_O_2_ showed the highest accumulation of ROS cancer cells.

### 3.6. Detection of Caspase Activity

**Figure 5:**
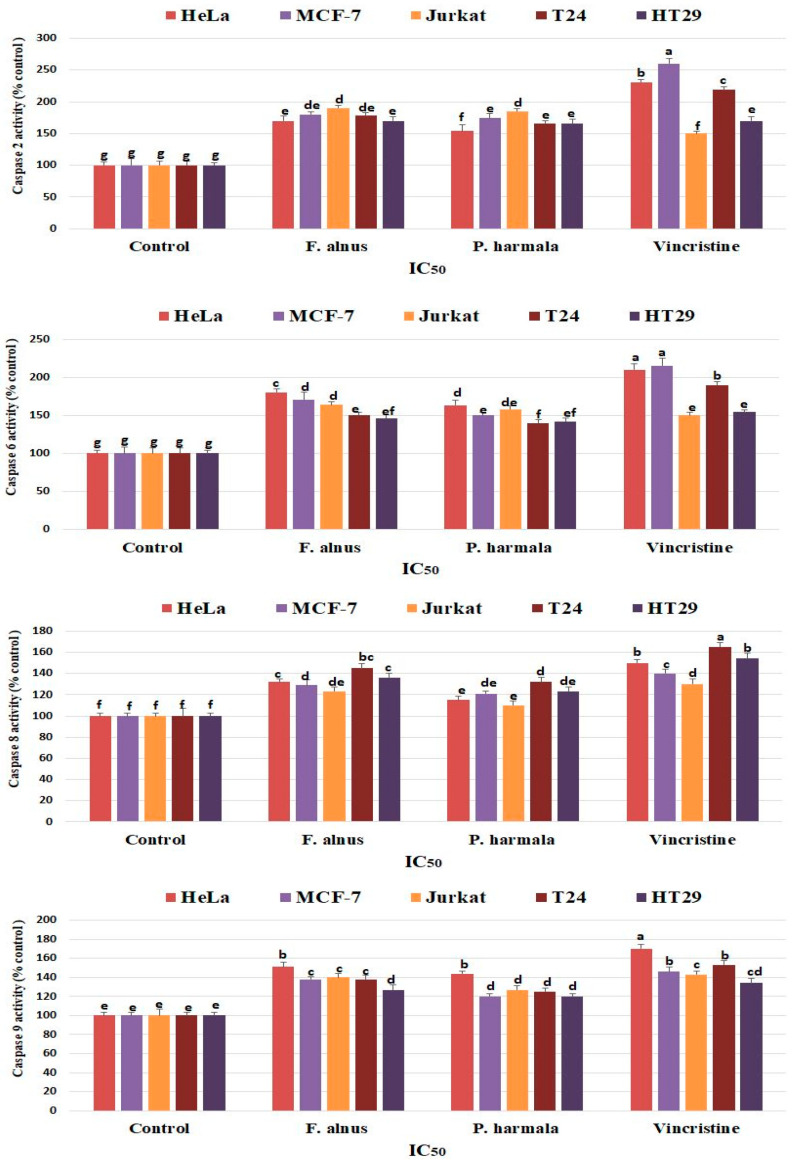
Enzyme activity of caspase 2, 6, 8 and 9 following treatment of different cancer cells with *F. alnus* and *P. harmala* leaf extracts (IC_50_). The results showed that increased caspase activity occurred after *F. alnus* and *P. harmala* treatment in all cancer cell lines compared to the control cells. The activity was expressed as a percentage (%) of control. The values are means ± SDs of three series of experiments. Different letters among columns indicate significant differences at *p ≤ 0.05*.

The effects of *F. alnus* and *P. harmala* leaf extracts on caspase 2, 6, 8 and 9 activities were studied in selected cancer cells (Figure 5). The results showed that increased caspase activity occurred after *F. alnus* and *P. harmala* treatment in all cancer cell lines compared to the control cells. In caspase 2, the treatment with *F. alnus* and *P. harmala* leaf extracts showed the highest activities in Jurkat cells. In caspase 6 and 9, the treatment with *F. alnus* and *P. harmala* leaf extracts showed the highest activities in HeLa cells. In caspase 8, the treatment with *F. alnus* and *P. harmala* leaf extracts showed the highest activities in T24 cells.

## 4. Discussion 

This is the first study exploring the polyphenolic compositions of *F. alnus* and *P. harmala*. The common raw material of *F. alnus* is the bark, which contain frangulins, anthraquinone glycoside derivatives, anthtaquinone monoglycosides (emodin) and glucofrangulins components [11,12]. In the current study, we revealed five phenolic acids and four flavonoids. Rosmarinic and chlorogenic acids were the main phenolic acids, while quercitrin and trifolin were the major flavonoids (Table 1). Recently, Nejabatdoust et al. [36] studied hydroalcoholic, ethanolic and methanolic bark extracts of *F. alnus* collected from Iran. They determined the total phenolic composition in the extracts and only identified 2,4-di-tert-butylphenol and butylated hydroxytoluene with the GC/MS analysis. They indicated no presence of phenolic acids and flavonoids. Maleš et al. [11] quantified the glucofrangulins and the phenolic compounds in Croatian *Rhamnus* and *Frangula* species. In the methanolic bark extracts of the Croatian *F. alnus* they estimated, using a spectrophotometric method, the total flavonoids, phenolic acids and total polyphenols components and their contents ranged from 0.05 to 0.08%, 1.21–1.44% and 5.57–8.30%, respectively. The variation of the polyphenolic composition of this species might be affected by an environmental factor such as light, which may influence the tannins in this species [15]. Indeed, high temperature and sunny conditions in Saudi Arabia influences the chemical composition of this species.

*P. harmala* is a species known by the presence of specific harmala alkaloids, which are at least 5.9% of the dry weight [37]. In the current study, the extracts of *P. harmala* contained four phenolic acids, with hydrocaffeic and protocatechuic acids as the major compounds. In addition, high amount of one flavonoid (cynaroside) were detected (Table 1). Sodaeizadeh et al. [38] studied the Iranian *P. harmala* using HPLC and determined seven phenolic acids in leaves and four phenolic acids in roots. 4-Hydroxybenzoic acid was the dominant compound some plant parts (leaves and roots), whereas caffeic acid was the highest in other parts such as the stems. In the current study, we investigated Arabian origin leaf extracts and characterized different phenolic acid components (only caffeic acid was a common compound between the two studies). We, additionally, confirmed hydrocaffeic, rosmarinic and protocatechuic acids. From flavonoids, Sharaf et al. [39] studied the leaf extracts of the Egyptian *P. harmala* and confirmed other compounds including peganetin, acacetin 7-0-rhamnoside, 7-0-[6”-O-glucosyl-2”-O-(3”‘-acetylrhamnosyl)glucoside, 7-O-(2”‘-0-rhamnosyl-2”-O-glucosylglucoside) and glycoflavone 2”‘-O-rhamnosyl-2”-O-glucosylcytisoside using TLC, and NMR methods. In the current study, and from our collection of widely distributed flavonoids, we tentatively determined only cynaroside (luteolin 7-glucoside). Previous investigation showed that environmental factors may influence the chemical composition of this species [40]. They reported elevated composition of vasicine, choline and sucrose in May as well as increases in betaine, lysine, 4-hydroxyisoleucine and proline in August. Furthermore, increases in phosphorylcholine, glucose, acetic acid and vasicinone were reported in December.

The antioxidant effects of *F. alnus* are mainly attributed to some leaves’ polyphenols including trifolin, *p*-coumaric acid, rosmarinic acid, quercetin, chlorogenic acid and ferulic acid. Trifolin is a kaempferol 3-galactoside flavonoid, and few studies detected this polyphenol in plants. Leaf extracts of *Zanthoxylum bungeanum* showed strong antioxidant effects in a previous study and were attributed to specific polyphenols such as trifolin (31.24  mg/g) [41]. In our study, *p*-coumaric acid showed noticeable antioxidant effects, which is in agreement with pervious investigations on other plants [42,43]. Rosmarinic acid antioxidant activities were comparable to antioxidant standards. In a previous investigation on the Saudi-origin *Artemisia abrotanum*, a strong antioxidant activity was detected and it was attributed to several polyphenols including rosmarinic acid [44]. The antioxidant activity of *P. harmala* is mainly attributed to polyphenols such as hydrocaffeic acid, cynaroside and protocatechuic acid. *Elsholtzia bodinieri* showed antioxidant effects attributed to specific flavonoids including cynaroside [45]. Protocatechuic acid is a phenolic acid commonly found in plants and has a strong antioxidant activity [45]. This activity is attributed to chelating metal ions and scavenging free radicals. Hydrocaffeic acid is not commonly found in plants as the caffeic acid. In the current study, we found high concentrations in the leaves of *P. harmala*. These high concentrations are associated with the high antioxidant effects. 

Most of the previous investigations on *F. alnus* focused on the bark, which is commonly used as laxative in herbal and alternative medicines. For example, the bark extract of *F. alnus* (Croatian origin) showed antioxidant effects attributed to emodin content [12]. From our knowledge, this is the first report confirming the antioxidant activities and polyphenolic composition of leaf methanolic extracts of *F. alnus*. The seed extracts of Indian-origin *P. harmala* showed high antioxidant properties in a previous investigation [17]. Previous investigation on Tunisian-origin *P. harmala* leaf extracts showed strong antioxidant and antibacterial activities [18]. However, no polyphenols were identified. Algerian-origin *P. harmala* leaf extracts showed bactericidal activities against *S. aureus* but no polyphenols were associated with this activity [19].

There were antiproliferative activities of *F. alnus* and *P. harmala* extracts against cancer cells (Table 3). *F. alnus* showed higher antiproliferative activities than *P. harmala.* In addition, there were antiproliferative activities when applying polyphenols such as quercitrin, cynaroside, trifolin, *p*-coumaric acid and rutoside against [22] cancer cells. Flow cytometry showed obvious apoptotic cell accumulation when applying *F. alnus* and *P. harmala* methanolic leaf extracts, as well as quercitrin, trifolin and cynaroside. In a previous investigation on Bosnian-origin *F. alnus*, a mild cytotoxic effect was found in the plant extracts against HeLa cancer cells but no activity was found when using the extracts of Serbian-origin plants [46]. No studies revealed a complete polyphenolic picture of this species. The antiproliferative and cytotoxic activities of *F. alnus* found here are related to the main polyphenols detected, such as quercitrin and trifolin. A previous investigation revealed that the quercetin flavonoid has antiproliferative activities against RAW264.7 cancer cell lines [47]. Other studies revealed that quercetin has cytotoxic activity against lung cancer cells [48]. Trifolin is a galactosideconjugated kaempferol that is formed by the kaempferol 3-ogalactosyltransferase and has apoptotic activity against lung cancer cells attributed to intrinsic and extrinsic pathways [49].

Previous investigations showed that *P. harmala* seeds and isolated compounds such as vasicinone, harmine, peganine and harmalacidine have antiproliferative and cytotoxic activities against Med-mek carcinoma, UCP-med sarcoma and Jurkat [20,21]. Harmine inhibited cell growth and vasicinone showed strong antiproliferating activity. The Chinese-origin *P. harmala* showed high amounts of 4-hydroxyisoleucine, asparagine, proline, lysine, vasicine and sucrose in the methanolic extracts of leaves [22]. However, no studies associated the leaf polyphenols with the cytotoxic activities of leaf extracts because larger interest was given for *P. harmala* seeds. In the current study, the antiproliferative and cytotoxic activities of *P. harmala* leaf extracts were mainly attributed to major polyphenols including cynaroside, hydrocaffeic acid and protocatechuic acid. Only a single previous investigation showed that cynaroside isolated from Turkish-origin *Teucrium chamaedrys* has antiproliferative activities against HeLa cells [50]. However, the current investigation is the first report studying the cytotoxic activities of cynaroside against HeLa, as well as other, cancer cells. Protocatechuic acid (3,4-dihydroxybenzoic acid) has anticancer effects against known cancer cells [51,52,53].

The caspase family proteases play an essential role in the apoptosis mechanism by controlling the pathway of apoptosis [54]. We found strong evidence that these caspase enzymes are activated in cells when treated with *F. alnus* and *P. harmala* leaf extracts. Caspases-8 and 9 are considered as large prodomains and initiator caspases, while caspase 6 is small prodomain [55]. In caspase-3 deficient cancer cells such as MCF-7, caspase 6, 8 and 9 could be involved in apoptosis [56]. Caspase 2 is not classified yet as an initiator or as an effector. However, it is involved in the cell death by activation within the p-53 protein with death domain PIDDosome [54]. From our knowledge, the effects of *F. alnus* and *P. harmala* leaf extracts on caspases activities have not been studied before.

## 5. Conclusions

The novel findings of this study can be summarized in the exploration of the polyphenol composition, and associated anticancer activities, of methanolic leaf extracts of *F. alnus* and *P. harmala* from natural populations in Saudi Arabia. Several polyphenols were identified by HPLC-DAD in the leaf extracts, including *p*-coumaric acid, rosmarinic acid, chlorogenic acid, ferulic acid, quercitrin, rutoside, quercetin and trifolin, in *F. alnus,* and hydrocaffeic acid, protocatechuic acid, caffeic acid, rosmarinic acid and cynaroside, in *P. harmala. F. alnus* and *P. harmala* leaf extracts showed antiproliferative and cytotoxic activities against cancer cells using MTT and flow cytometry assays. These anticancer activities were attributed to the polyphenolic composition of leaves including quercitrin, trifolin and cynaroside, which resulted in necrotic cell accumulation during apoptotic phases. *F. alnus* and *P. harmala* showed strong antioxidant effects attributed to the polyphenolic composition of leaves including quercitrin, trifolin, *p*-coumaric acid, rutoside, rosmarinic acid, quercetin, chlorogenic acid and ferulic acid in *F. alnus* and cynaroside, hydrocaffeic acid and protocatechuic acid in *P. harmala.* These antioxidant effects were associated with reduced ROS production in treated cells compared to controls. This is the first study investigating the antioxidant activities of hydrocaffeic acid and showing strong antioxidant activities. Finally, this is the first study revealing the activation of caspase family proteases in cancer cells by *F. alnus* and *P. harmala* leaf extracts.

## Figures and Tables

**Table 1:** The polyphenolic composition of *F. alnus* and *P. harmala* leaf extracts (mg/100 g DW ± SD).

	Phenolic Compounds	(mg/100 g DW ± SD)
***Frangula alnus***	Chlorogenic acid (1)	126.8 ± 2.5
	Caffeic acid (2)	33.8 ± 2.1
	*p*-Coumaric acid (3)	41.4 ± 2.7
	Ferulic acid (4)	44.0 ± 3.4
	Rutoside (Quercetin 3-rutinoside) (5)	283.4 ± 23.9
	Unknown compound (6)	1072.4 ± 94.7
	Quercetin (7)	115.1 ± 16.4
	Rosmarinic acid (8)	269.5 ± 14.7
	Quercitrin (9)	1132.3 ± 76.2
	Trifolin (Kaempferol 3-galactoside) (10)	914.3 ± 20.7
***Peganum harmala***	Caffeic acid (2)	20.6 ± 1.8
	Rosmarinic acid (8)	18.9 ± 0.3
	Protocatechuic acid (11)	51.6 ± 6.8
	Hydrocaffeic acid (12)	199.4 ± 12.9
	Unknown compound (13)	503.8 ± 47.3
	Unknown compound (14)	254.0 ± 28.1
	Cynaroside (Luteolin 7-glucoside) (15)	713.5 ± 88.0

DW:dry weight.

**Table 2:** Antioxidant activities of *F. alnus* and *P. harmala* and identified polyphenols (quercitrin, trifolin, *p*-coumaric acid, cynaroside, rutoside, rosmarinic acid, quercetin, protocatechuic acid, chlorogenic acid, hydrocaffeic acid and ferulic acid) using different assays (expressed as IC_50_ in µg/mL).

	*β*-Carotene-Bleaching Assay (IC_50_, µg/mL)	DPPH (IC_50_, µg/mL)	FRAP (IC_50_, mM TEAC/g extract)
***F.alnus***	18.3 ± 2.3 b	14.1 ± 1.2 b	23.1 ± 1.3 b
***P. harmala***	26.8 ± 2.9 a	21.5± 2.3 a	32.4 ± 3.5 a
**Quercitrin**	26.0 ± 1.9 a	21.5 ± 1.5 a	32.6 ± 3.1 a
**Trifolin**	6.9 ± 0.3 d	5.6 ± 0.2 d	8.6 ± 0.3 e
***p*-Coumaric acid**	4.0 ± 0.3 e	3.8 ± 0.1 e	4.3 ± 0.1 g
**Cynaroside**	6.6 ± 0.3 de	5.8 ± 0.3 d	8.3 ± 0.3 e
**Rutoside**	17.5 ± 0.7 b	14.3 ± 0.9 b	19.3 ± 1.5 c
**Rosmarinic acid**	3.0 ± 0.2 e	2.5 ± 0.3 f	3.4 ± 0.3 g
**Quercetin**	6.3 ± 0.2 e	5.6 ± 0.3 d	8.0 ± 0.3 e
**Chlorogenic acid**	5.0 ± 0.5 ef	4.1 ± 0.2 e	6.7 ± 0.5 f
**Ferulic acid**	9.8 ± 0.3 c	8.0 ± 0.3 c	11.7± 1.1 d
**Hydrocaffeic acid**	4.5 ± 0.1 f	4.1 ± 0.1 e	5.3 ± 0.1 f
**Protocatechuic acid**	9.2 ± 0.1 c	7.3 ± 0.1 c	11.2 ± 0.1 d
**BHT**	3.3 ± 0.1 g	2.9 ± 0.1 f	-
**Trolox**	-	-	3.2 ± 0.1g

Values are expressed as mean ± standard deviation. TEAC: Trolox equivalent antioxidant capacity. Within column different letters indicate significant differences (*p* ≤ 0.05). FRAP: ferric reducing antioxidant power, DPPH: 2,2-diphenyl-1-picrylhydrazyl.

**Table 3:** Antiproliferative activity of *F. alnus* and *P. harmala* methanolic extracts, quercitrin, cynaroside, trifolin, *p*-coumaric acid, rutoside, quercetin, chlorogenic acid, ferulic acid, hydrocaffeic acid, and protocatechuic acid [IC_50_ (µg mL^-1^)] against different cancer cells.

	HT-29 *	HeLa	MCF-7	Jurkat	HEK-293
***F. alnus***	31.53 ± 2.1 c	28.45 ± 1.3 d	39.64 ± 3.3 b	43.75 ± 3.8 d	˃400
***P. harmala***	49.05 ± 3.2 b	43.86 ± 2.7 b	58.64 ± 4.2 a	59.53 ± 4.2 b	˃400
**Rutoside**	19.1 ± 0.5 fe	4.1 ± 02 f	5.74± 0.6 fg	4.7 ± 0.2 e	˃400
**Cynaroside**	7.9 ± 0.6 f	4.8 ± 0.5 f	25.97 ± 1.7 e	42.32 ± 2.1 d	˃400
**Chlorogenic acid**	14.11 ± 2.6 e	4.1 ± 0.5 d	37.25 ± 3.9 c	40.53 ± 2.1 d	˃400
**Ferulic acid**	21.32 ± 7.1 d	50.35 ± 3.9 a	41.32 ± 3.5 b	38.53 ± 3.8 d	˃400
**Quercetin**	7.50 ± 0.8 f	4.9 ± 0.7 f	22.53 ± 1.5 e	39.63 ± 2.3 d	˃400
**Quercitrin**	20.32 ± 1.8 d	17.3 ± 1.2 e	45.23 ± 4.1 b	68.42 ± 3.8 a	˃400
**Trifolin**	18.9 ± 0.9 d	16.1 ± 1.7 e	28.21 ± 1.6 d	45.31 ± 2.1 c	˃400
***p*-Coumaric acid**	8.2 ± 0.3 f	6.3 ± 0.6 f	18.5 ± 2.1 f	33.5 ± 2.7 d	˃400
**Protocatechuic acid**	97.42 ± 4.1 a	38.19 ± 3.5 c	19.31 ± 2.9 f	48.31 ± 3.1 c	˃400
**Hydrocaffeic acid**	19.3 ± 1.9 d	15.42 ± 1.3 e	23.1 ± 2.3 e	39.12 ± 2.5 d	˃400
**Vinblastine sulfate**	15.3 ± 0.2 e	2.2 ± 0.01 f	‒	0.14 ± 0.02 f	43.5 ± 2.5
**Taxol**	‒	‒	0.06 ± 0.005 h	‒	‒
**Vincristine**	8.4 ± 0.4	4.64 ± 1.5	0.4 ± 0.07	90.1 ± 3.2	46.5 ± 0.3

Different letters within a column indicate significant differences at *p ≤* 0.05.
